# OPTIMAL, an occupational therapy led self-management support programme for people with multimorbidity in primary care: a randomized controlled trial

**DOI:** 10.1186/s12875-015-0267-0

**Published:** 2015-05-12

**Authors:** Jess Garvey, Deirdre Connolly, Fiona Boland, Susan M Smith

**Affiliations:** Department of Occupational Therapy, Trinity College Dublin, Trinity Centre, St James’s Hospital, Dublin 8, Ireland; HRB Centre for Primary Care Research, Department of General Practice, Royal College of Surgeon’s in Ireland, Beaux Lane House, Lower Mercer Street, 2, 123 St Stephen’s Green, Dublin 2, Ireland

**Keywords:** Occupational therapy, Multimorbidity, Randomised controlled trial, Self-management

## Abstract

**Background:**

We investigated the effectiveness of an occupational therapy led self-management support programme, OPTIMAL, designed to address the challenges of living with multiple chronic conditions or multimorbidity in a primary care setting.

**Methods:**

Pragmatic feasibility randomised controlled trial including fifty participants with multimorbidity recruited from family practice and primary care settings. OPTIMAL is a six-week community-based programme, led by occupational therapy facilitators and focuses on problems associated with managing multimorbidity. The primary outcome was frequency of activity participation. Secondary outcomes included self-perception of, satisfaction with and ability to perform daily activities, independence in activities of daily living, anxiety and depression, self-efficacy, health-related quality of life, self-management support, healthcare utilisation and individualised goal attainment. Outcomes were collected within two weeks of intervention completion.

**Results:**

There was a significant improvement in frequency of activity participation, measured using the Frenchay Activities Index, for the intervention group compared to the control group (Adjusted Mean Difference at follow up 4.22. 95% Confidence Interval 1.59-6.85). There were also significant improvements in perceptions of activity performance and satisfaction, self-efficacy, independence in daily activities and quality of life. Additionally, the intervention group demonstrated significantly higher levels of goal achievement, following the intervention. No significant differences were found between the two groups in anxiety, depression, self-management scores or healthcare utilisation.

**Conclusions:**

OPTIMAL significantly improved frequency of activity participation, self-efficacy and quality of life for patients with multimorbidity. Further work is required to test the sustainability of these effects over time but this study indicates that it is a promising intervention that can be delivered in primary care and community settings.

**Trial registration:**

Trial Number: ISRCTN67235963

## Background

Multimorbidity is now considered the norm, rather than the exception, in primary care settings [1,2]. It is an independent predictor of adverse outcomes, including poor quality of life (QoL), mortality and disability [3]. It is also linked to psychological distress, lower levels of functioning, financial difficulties, restrictions in work, leisure and social activities, multiple symptoms and increased healthcare utilization (HCU) [4-7]. Qualitative research indicates that individuals with multimorbidity have concerns over functional limitations and interference with daily routines [8]. Despite this, there is limited evidence regarding the potential effectiveness of interventions designed to improve outcomes in multimorbidity. A Cochrane review of such interventions found ten randomised controlled trials and results were mixed. The review suggested that interventions targeting functional limitations that are common across conditions had potential to improve outcomes [9]. Occupational therapy was identified as a discipline that incorporates the skills and techniques to address these difficulties [10].

We used the UK Medical Research Council Framework for the design and evaluation of complex interventions to improve health outcomes to develop the intervention and after two initial pilot studies, the intervention was refined to a six-week occupation-based self-management programme, OPTIMAL, specifically designed to target individuals with multimorbidity [11]. Self-management, sometimes referred to as self-care has been defined as the actions taken by individuals to lead a healthy lifestyle, to meet their needs and to care for their long-term conditions to prevent further future illness [12]. Full details of the OPTIMAL programme content and delivery have been described previously following the initial development and pilot study [11]. The programme was based on the Stanford Chronic Disease Self-Management Programme (CDSMP) [13] with the key adaptations being an occupational therapy focus, groups being professionally led and a clear focus on the specific challenges of multimorbidity identified from the qualitative literature in this area [8]. We adapted this programme based on the need to develop effective interventions for patients with multimorbidity as existing evidence suggests that the CDSMP has modest effects [14], particularly when delivered in settings outside the USA [15]. The intervention was designed to be professionally led, with the aim of harnessing the effective elements of other successful professional-led interventions such as cardiac and pulmonary rehabilitation [16]. The theoretical underpinning for the OPTIMAL Intervention is Bandura’s Theory of Self-Efficacy. Bandura defined self-efficacy as *“…peoples judgment of their capacity to organise and execute courses of action required to attain designated types of performances”* (pg. 391) [17]. In the context of the OPTIMAL intervention, the proposed improvement in self-efficacy would be expected to enhance self-management and confidence, which would in turn enable patients to manage their symptoms and have improved performance of daily activities and improved well-being. Patients with multimorbidity may particularly benefit from such programmes as they have been shown to have low levels of self-efficacy and poor quality of life, both of which worsens with increasing numbers of conditions. This likely relates to the increasing complexity of managing additional conditions and the increasing burden of symptoms and of treatment [18]. These considerations have driven intervention design and development.

We aimed to undertake a pragmatic feasibility randomised controlled trial to determine the effectiveness of OPTIMAL for increasing activity participation in individuals with multimorbidity.

## Methods

We undertook a pragmatic feasibility randomised controlled trial using the CONSORT Guidelines to ensure accurate and complete reporting of the design, conduct and analysis of the study (www.consort-statement.org). The study was approved by the Research Ethics Committee of Trinity College Dublin (Study reference: 21 November, 2012).

### Setting and patients

Participants with multimorbidity were identified opportunistically during routine encounters by their Family Practitioner and other primary care clinicians and referred to the occupational therapists running the programme until a sufficient number of participants were recruited. Contextual details of the Irish health system are provided in Table 1. Given the pragmatic nature of this feasibility trial there was no clear denominator of all eligible patients. Patients were recruited across three community care areas in which participating occupational therapists were based and these areas cover a population of approximately 67,000 people. Clinicians (family practitioners or any other primary care clinicians in the areas) were emailed with information and study inclusion criteria and encouraged to refer any eligible patients over a three-month period (December 2012 to February 2013). This replicates how an intervention such as OPTIMAL would be offered and delivered in clinical practice but means it is not possible to calculate the numbers of potentially eligible patients in the population. However, given the prevalence of multimorbidity this would be far higher than the numbers that were needed for this feasibility trial. The following inclusion criteria were applied: age over 18 years; patients with two or more chronic conditions and a minimum of four repeat medications. There has been much discussion in the literature on definitions of multimorbidity with variation in criteria applied across studies [19]. We used a broad inclusive definition of two chronic conditions applying the WHO definition of a chronic condition which is a condition likely to persist for longer than six months and need life long intervention [20]. However, as the prevalence studies suggest this represents the norm in older population so we also added matched criteria that patients should be receiving at least four repeat prescriptions. This was to ensure that individuals with more manageable combinations of relatively asymptomatic conditions, for example hyperlipidaemia and well-controlled hypertension were not targeted. For pragmatic reasons this also enabled clinicians to focus on referring individuals who were more likely to need support managing their conditions. We excluded patients who were unable to travel to the community centre where groups were delivered or who had participated in the initial pilot study and referring practitioners were aware of this exclusion criterion. Written consent was obtained from all participants who took part in the trial. The trial began in November 2012 and ended in June 2013.

**Table 1:** **Key features of healthcare systems in the Republic of Ireland**

**Irish healthcare system**	**In the Irish healthcare system there is mixed public and private funding. Primary health care is free through the Primary Care Reimbursement Scheme (PCRS) only to those judged less able to pay and covers approximately 35% of the population. Registration with a family practice is required only if the patient belongs to the PCRS. PCRS supports the delivery of primary healthcare by providing reimbursement services to primary care contractors for the provision of health services to members of the public in their own community. Patients not eligible for this scheme pay approximately €50 ($69) per visit.**
	**Primary care in Ireland is an inter-disciplinary team-based approach. Primary care services are provided by family practitioners, nurses, health care assistants, physiotherapists, occupational therapists, social workers, speech and language therapists and pharmacists. The model of primary care supported by government but not yet fully implemented. The proposed model emphasises a change from secondary care to more appropriate primary care services, to provide a single point of entry for all health and personal social services.**

### Randomisation and allocation concealment

This study was an individually randomised trial, where participants were randomly allocated to receive the intervention or to remain on the waiting list and receive usual care [21]. Patients were randomised when baseline data collection was complete. The randomisation and allocation were carried out remotely by a statistician, independent of the trial management team with no involvement in patient recruitment, using a computer generated sequence. The researcher informed participants of their group assignment one to two weeks prior to intervention commencement by telephone. It took between four to eight weeks to fill a group.

### Intervention

The intervention was the six-week OPTIMAL programme and intervention elements are summarised in Table 2. It was delivered in three different primary care centres in local communities near where patients lived. In total, three OPTIMAL programmes were delivered, staggered over a three month period. The overall aims of OPTIMAL are to improve performance, satisfaction and frequency of activity participation, increase self-efficacy in managing multimorbidity, improve quality of life, reduce anxiety and depression, and improve multimorbidity self-management skills. The OPTIMAL programme is professionally led and facilitated by occupational therapists but incorporates elements of peer support available through the group format. Prior to programme delivery OPTIMAL facilitators received two training sessions from the study team (DC and JG) and a group leader manual to standardise programme delivery. Full details of the course content are available in the published pilot study [11]. In summary, the group meetings were held weekly over a six-week period with each meeting lasting three hours. The meetings were led by a local community based occupational therapist (OT) working with the research OT (JG). The sessions covered the following topics: Fatigue management; healthy eating; maintaining physical activity (delivered by a community physiotherapist); maintaining mental health; managing medications (delivered by a pharmacist) and communicating effectively with health professionals. Individual goal setting was a key element with goals discussed and revised at each session.

**Table 2:** **Elements of the OPTIMAL Intervention**

**Breakdown of OPTIMAL Programme**	**OPTIMAL has the following elements:**
	**1. Weekly group meetings for a six-week period held in local community health centres**
	**2. Occupational Therapy focus**
	**3. Peer support**
	**4. Goal setting and prioritization based on patient preferences**
	**OT interventions to support patient self-management used in the groups include:**
	**• Self-management**
	**• Fatigue and energy management**
	**• Managing stress and anxiety and maintaining mental health and well-being**
	**• Keeping physically active**
	**• Healthy eating**
	**• Managing medications**
	**• Effective communication strategies**
	**• Goal setting**
	**One of the weekly sessions incorporates education on physical activity delivered by a physiotherapist and another incorporates medicines management, delivered by a pharmacist**

### Control

All 50 participants underwent assessment and baseline data collection prior to randomisation. Patients allocated to control were placed on a waiting list and were invited to attend an OPTIMAL course following trial completion in their local Occupational Therapy Department.

### Outcomes

#### Primary outcome

The Frenchay Activities Index (FAI) was used to measure the primary outcome, frequency of activity participation [22]. The FAI was chosen as the primary outcome as previous research has indicated that those with multimorbidity engage less frequently in productive and leisure activities despite having the ability to do so [23]. One of the primary objectives of the programme therefore is to increase frequency of activity engagement. Although originally designed to measure changes in activities after stroke, it has been identified as valid and reliable with a variety of health conditions and in community dwelling populations. The score range is 0–45, with higher scores indicating higher frequency of activity participation. It is divided into three categories of activities (domestic chores, leisure/work and outdoor activities), with each category scoring 0–15 [22].

### Secondary outcomes

Patient-based secondary outcome measures included: the Canadian Occupational Performance Measure (COPM) [24]; the Nottingham Extended Activities of Daily Living (NEADL) [25]; the Hospital Anxiety and Depression Scale (HADS) [26]; the Stanford Chronic Disease Self-Efficacy 6-item Scale (SSE) [27]; the EQ-5D [28]; the Health Education Impact Questionnaire (HeiQ) [29]; and Goal attainment scaling (GAS) [30]. Health care utilisation including number of family practitioner visits, practice nurse visits and hospital admissions were collected directly from family practice medical records.

### Sample size

Sample size calculation was based on the primary outcome, the Frenchay Activities Index. Improvements of four points in FAI total scores have been reported as clinically significant [31]. The pilot study revealed baseline FAI scores of 26.5 (SD 3.23) [11]. To improve these scores by 4 points, with 90% power, allowing for a 20% loss to follow up, a total sample of 34 participants was required. We aimed to recruit 60 participants in total, 30 in each arm with three groups running for intervention group participants in the three primary care centres involved, and 10–12 patients per group as some variation in group attendance over the programme was anticipated.

### Data collection

Data were collected two weeks prior and two weeks post intervention in primary care centres or participants’ homes, depending on participants’ preferences. All outcome measures were conducted face to face with participants. Baseline data were collected prior to allocation to minimise ascertainment bias. Due to the nature of the intervention it was not possible to blind follow up data collection.

### Data analysis

For the primary outcome, a multiple linear regression model was used to examine the relationship between FAI follow-up scores and group allocation (intervention or control). Baseline FAI scores, age, sex, marital status, occupational status and number of chronic conditions were controlled for as potential confounding variables [4,32-35]. P-values <0.05 were deemed statistically significant. An intention to treat (ITT) analysis was conducted which included only individuals with complete baseline and follow-up data, i.e. complete case analysis. An additional sensitivity analysis was conducted imputing missing values using the method of ‘last observation carried forward’ [36].

The differences for all FAI subscales and secondary outcomes between baseline and follow-up for the intervention and control group were examined. For continuous outcomes the mean and standard deviation were calculated and for categorical outcomes frequencies and percentages were calculated. Shapiro-Wilk tests of normality were conducted to determine appropriate parametric and non-parametric statistical tests. Independent sample t-tests, Mann Whitney U tests and proportions tests were selected accordingly. Linear regression models, adjusting for baseline values, age, sex, marital status, occupational status and number of chronic conditions were used where appropriate. The Bonferroni correction was used to adjust for multiple comparisons for the secondary outcome measures. Including all secondary outcomes measures and subscales, 23 tests were run. We multiplied all the unadjusted p-values by 23 and compared these adjusted p-values to 0.05.

## Results

### Patient flow and characteristics

Sixty-three individuals were referred to the programme within the study time frame, which was limited as this work was undertaken as part of a postgraduate Research Masters degree (JG). Patients were referred by family practitioners and other primary care clinicians from three defined Primary Care Network areas in Dublin, covering a population of approximately 67,000 people. Thirteen participants dropped out prior to baseline data collection due to lack of interest, busy schedules or recent hospitalisations. Hence, fifty participants completed baseline assessment and were included in the study.

**Figure 1:**
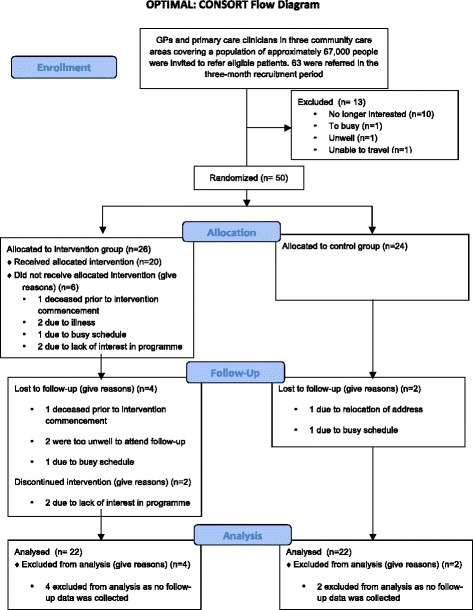
Flow diagram of the progress of the intervention and control group throughout the RCT.

Figure 1 presents an overview of participant follow up during the trial. Of the fifty participants recruited into the study, 44/50 (88%) had complete baseline and follow-up data sets. In total, 6/50 (12%) participants were lost to follow-up; four from the intervention group and two from the control group. The majority of the intervention group (20/26: 76%) attended three or more of the six sessions but 6/26 (13%) never attended any session.

The median age of participants was 66 years and the majority were married, living with a family member and retired. Participants had a median number of four conditions and were taking a median number of eight medications. In total, 43 chronic conditions were identified, the most common being arthritis, congestive cardiac failure, diabetes, depression and hypertension (Table 3).

**Table 3:** **Summary of participant characteristics**

	**Intervention group (n = 26)**	**Control group (n = 24)**
**Median Age (Range)**	65 (50–83)	67.5 (42–84)
**Gender**		
Male	9 (34.6%)	9 (37.5%)
Female	17 (65.4%)	15 (62.5%)
**Median no. of Conditions (Range)**	4 (2–9)	5 (2–9)
**Median no. of Medications (Range)**	7 (4–16)	11 (4–21)
**Marital Status**		
Single	5 (19.2%)	7 (29.2%)
Married	9 (34.6%)	10 (41.7%)
Widowed	5 (19.2%)	4 (16.7%)
Divorced/Separated	7 (26.9%)	3 (12.5%)
**Living Situation**		
Alone	10 (38.5%)	11 (45.8%)
With Partner/Family Member	16 (61.5%)	13 (54.3%)
**Employment Status**		
Employed	3 (11.5%)	0 (0%)
Unemployed	8 (30.7%)	8 (33.3%)
Retired	15 (57.7%)	16 (66.7%)
**Educational Status**		
Did not complete secondary education	19 (73.1%)	17 (70.8%)
Completed secondary education	7 (26.9%)	7 (29.2%)

Unless otherwise stated the figures are numbers and percentages.

### Primary outcome: frequency of activity participation

The multiple linear regression model, having adjusted for baseline values, age, sex, marital status, occupational status and number of chronic conditions, indicated significant differences in the total FAI scores between the intervention and control group at follow up (Adjusted MD at follow up 4.22 (95% CI 1.59-6.85), (see Table 4). The Adjusted R Squared value was 0.705, indicating that 70.5% of the variation in FAI outcomes was explained by group allocation. The only significant difference in FAI subscales, between the intervention and control group, was the domestic subscale (p < 0.01).

**Table 4:** **A comparison of FAI baseline and follow-up scores**

	**Intervention group (n = 22)**		**Control group (n = 22)**			
	**Mean (SD)**					
**FAI Scores**	**Baseline**	**Follow-up**	**Baseline**	**Follow-up**	**Difference (95% CI) at follow-up**	**P-value**
**Domestic**	8.6 (4.57)	10.6 (3.82)	9.9 (3.18)	9.1 (4.16)	2.77 (0.70 – 3.31)	<0.01
**Work/Leisure**	4.6 (3.55)	5.6 (1.99)	3.5 (2.61)	3.3 (2.10)	1.18 (0.17 – 2.53)	0.12
**Outdoor**	8.1 (2.21)	8.7 (3.21)	6.6 (2.97)	6.6 (3.02)	0.64 (0.69 – 1.97)	0.37
**Total**	21.3 (7.87)	24.9 (7.37)	19.8 (6.46)	18.9 (7.24)	4.22 (1.59 – 6.85)*	<0.01*

*Adjusted for baseline FAI values, age, sex, marital status, occupational status and number of chronic conditions.

FAI: range 0 – 45 (total), 0 – 15 (subscales). Higher scores = greater occupational performance.

### Secondary outcomes

There were significant differences in a range of secondary outcomes (see Table 5). Having adjusted for baseline values, age, sex, marital status, occupational status and number of chronic conditions, there was a significant difference between the intervention and control groups for the COPM-P (p = 0.02), COPM-S (p = 0.02), NEADL total score (p = 0.02), SSE (p = 0.02) and EQ-VAS (p = 0.02). No significant difference was seen for the HADS at follow-up (p = 0.49). Health care utilisation data revealed high levels of service utilisation, particularly family practice services. No significant differences were identified in HCU between the two groups, however, the follow-up time period was very short and the study was not powered to detect differences. The positive and active engagement in life domain was significantly higher for the intervention group than for the control group (p = 0.04) in the HeiQ scores. No significant findings were identified for the other seven domains.

**Table 5:** **A comparison of baseline and follow-up values for the intervention and control groups for secondary outcomes**

		**Intervention group (n = 22)**		**Control group (n = 22)**		**P-value** ^**♮**^
		**Mean (SD)**				
		**Baseline**	**Follow-up**	**Baseline**	**Follow-up**	
**COPM**	**Performance**	4.11 (1.4)	5.77 (1.83)	4.37 (1.28)	4.1 (1.35)	0.02*
	**Satisfaction**	3.16 (1.88)	5.57 (1.99)	3.12 (1.69)	3.42 (1.88)	0.02*
**NEADL**	**Mobility**	11.36 (4.03)	13.36 (4.04)	12.32 (3.90)	11.36 (4.49)	0.10
	**Kitchen**	12.59 (3.95)	13.55 (2.48)	13.64 (2.87)	13.09 (2.78)	0.50^♯^
	**Domestic**	9.45 (3.99)	10.18 (3.99)	9.55 (4.03)	8.18 (3.96)	0.20^♯^
	**Leisure**	10.14 (3.5)	10.09 (3.58)	9 (3.32)	8.09 (3.37)	0.99^♯^
	**Total**	43.09 (12.41)	47.18 (11.87)	44.45 (10.78)	40.73 (10.71)	0.02*
**HADS**	**Anxiety**	9.77 (5.07)	9.50 (4.71)	10.05 (5.00)	9.09 (4.96)	0.99
	**Depression**	6.68 (3.75)	6.32 (4.19)	6.82 (3.22)	7.82 (3.83)	0.99
	**Total**	16.68 (8.32)	15.59 (8.31)	17.09 (6.88)	16.68 (8.16)	0.49*
**SSE**		5.53 (1.88)	6.79 (1.51)	5.84 (2.04)	5.32 (1.92)	0.02*
**EQ-VAS**		49.86 (22.89)	65.73 (20.18)	54.27 (20.79)	50.50 (16.30)	0.02*
**HeiQ**	**1. Health-directed behaviour**	2.81 (0.74)	3.04 (0.69)	2.74 (0.73)	2.73 (0.81)	0.99
	**2. Positive and active engagement in life**	2.59 (0.61)	2.93 (0.63)	2.57 (0.44)	2.62 (0.56)	**0.04**
	**3. Emotional well-being**	2.07 (0.71)	2.35 (0.70)	2.11 (0.73)	2.23 (0.70)	0.99
	**4. Self-monitoring and insight**	3.12 (0.34)	3.25 (0.39)	2.98 (0.46)	2.97 (0.44)	0.99
	**5. Constructive attitudes and approaches**	2.98 (0.67)	3.01 (0.62)	3.06 (0.48)	2.95 (0.41)	0.99
	**6. Skill and technique acquisition**	2.84 (0.49)	3.04 (0.50)	2.83 (0.59)	2.78 (0.34)	0.56
	**7. Social integration and support**	2.85 (0.76)	3.01 (0.75)	2.75 (0.79)	2.84 (0.57)	0.99
	**8. Health service navigation**	3.09 (0.49)	3.15 (0.46)	3.12 (0.65)	3.05 (0.51)	0.99
**HCU**	**GP visits**	3.32 (2.65)	3.16 (3.35)	2.65 (1.87)	1.70 (1.45)	0.99
	**PN visits**	0.26 (0.45)	0.26 (1.15)	0.35 (0.59)	0.25 (0.44)	0.99^♯^
	**Hosp Admissions**	0.05 (0.23)	0.21 (0.42)	0.10 (0.31)	015. (0.37)	0.99^♯^

The Bonferroni method was used to account for multiple comparisons.

*Adjusted for baseline values, age, sex, marital status, occupational status and number of chronic conditions.

^♯^Mann Whitney U Test.

^♮^P values adjusted for bonferroni correction.

The results relating to health related QoL showed a trend towards improvement in a number of EQ-5D domains (mobility, usual activities, anxiety/depression) in the intervention group (see Table 6). However, having adjusted for multiple comparisons no significant differences between the intervention and control group were seen for any of the domains.

**Table 6:** **A comparisons of baseline and follow-up values for the intervention and control groups for EQ5D outcomes in the differences between baseline**

**EQ-5D dimension**	**Intervention group (n = 22)**		**Control group (n = 22)**	
	**Frequency (%)**			
	**Baseline**	**Follow-Up**	**Baseline**	**Follow-Up**
**Mobility**				
**• No Problems**	4 (18.2%)	6 (27.3%)	5 (22.7%)	6 (27.3%)
**• Moderate Problems**	18 (81.8%)	16 (72.7%)	17 (77.3%)	16 (72.7%)
**Self-Care**				
**• No Problems**	13 (59.1%)	14 (63.6%)	13 (59.1%)	16 (72.7%)
**• Moderate Problems**	9 (40.9%)	8 (36.4%)	9 (40.9%)	6 (27.3%)
**Usual activities**				
**• No Problems**	5 (22.7%)	9 (40.9%)	5 (22.7%)	6 (27.3%)
**• Moderate Problems**	17 (77.3%)	13 (59.1%)	17 (72.7%)	16 (72.7%)
**Pain/Discomfort**				
**• No Problems**	4 (18.2%)	3 (13.6%)	1 (4.5%)	3 (13.6%)
**• Some Problems**	18 (81.8%)	19 (63.6%)	21 (45.5%)	19 (86.4%)
**Anxiety/Depression**				
**• No Problems**	4 (18.2%)	8 (36.4%)	4 (18.2%)	4 (18.2%)
**• Moderate Problems**	18 (81.8%)	14 (63.6%)	18 (81.8%)	18 (81.8%)

Goal Attainment Scoring (GAS) was used only with intervention group participants and significant differences were found between baseline and follow-up (p ≤ 0.01). Results showed that 19/20 participants had significant levels of achievement in their identified goals. Commonly cited goals included improving fitness levels, losing weight, increasing confidence levels, improving diet and developing a more structured, daily routine. The median number of goals set was three.

## Discussion

This study showed that OPTIMAL, an occupational therapy–led self-management programme, is effective in improving activity participation and performance, and leads to improvements in self-efficacy, health related quality of life and goal attainment. OPTIMAL also increased participants’ frequency of engagement in daily activities such as household chores and meal preparation. These improvements have the potential to improve outcomes for patients through more effective management of long-term conditions. To our knowledge, it is the first fully professionally led self-management programme designed specifically for patients with multimorbidity rather than the more general concept of self-management for chronic conditions. This is conceptually different due to the specific focus on improving activity participation and functional abilities of participants as identified in qualitative studies. It also addresses generic self-management knowledge and skills across different conditions and the need for prioritisation of health problems through techniques such as goal setting. Existing Chronic Disease Self-Management Programmes focus on living with chronic conditions and generally include people with single conditions and have presented sub-group analyses on outcomes in participants with multimorbidity attending the same programmes [13,37]. Qualitative work with patients and practitioners suggest that multimorbidity, particularly for patients with higher numbers of conditions as included in this study, has additional challenges especially around treatment burden and the need to prioritise management of health problems based on patient preferences [38]. This study matches the eligibility criteria for inclusion in the planned update of the Cochrane Review of Interventions to Improve Outcomes for Patients with Multimorbidity and will add to the growing evidence base needed to support multimorbidity management [37].

OPTIMAL has a strong focus on improving and increasing activity participation. Areas identified as problematic for people with multimorbidity are discussed including, fatigue, anxiety, communicating with health professional and medication management [4,7]. Through active group discussion, participants examine how these issues can impact on activity performance and participation. The facilitators provide practical strategies on how to maintain participation in valued activities. There are also important inputs from a physiotherapist and pharmacist on managing physical activity and medications. Weekly goal-setting is another key aspect of OPTIMAL and each week participants set realistic and achievable goals to work on over the following week. Goal-setting has been shown in previous studies to be an effective mechanism to enhance self-management strategies [39,40].

There were significant differences between intervention and control groups in self-efficacy which is defined as the confidence in capacity to undertake complex steps required to effectively self-manage chronic conditions. Self-efficacy has been suggested as one of the main underpinnings of successful self-management programmes [41,42]. There is a potential bidirectional relationship here that as individuals increase their frequency of activity participation, their confidence in their abilities may improve and vice versa. There were also trends in improvements in health related quality of life. Research has highlighted the importance of control and choice on quality of life [43,44]. Giving participants autonomy to choose not only empowers individuals to make decisions about their own health management, but reflects current policy for health services to be person-centred [45,46].

On referral to the study 12% of the sample had a confirmed diagnosis of anxiety. However, baseline Hospital Anxiety and Depression Scale scores revealed 36% of the overall sample presented with “caseness” levels on the anxiety scale. While one of the weekly OPTIMAL sessions focuses on stress and anxiety management, this may not be enough to address these challenging symptoms. As discussed, only one of the eight Health Education Impact Questionnaire domains was found to be significant. Although this questionnaire was developed specifically for self-management programmes these findings may indicate it does not reflect the aims of OPTIMAL.

There were no significant differences in healthcare utilisation between the two groups, which likely reflects the short follow-up period and lack of power to detect such changes. To date, studies investigating the impact of self-management programmes have reported inconsistent findings on healthcare utilisation [47,48]. When improvements were noted, minimum follow-up was generally six months. However, healthcare utilisation is a complex outcome and it may be appropriate for it to increase or decrease depending on baseline utilisation. The programme content of OPTIMAL includes the development of effective communication skills and having the confidence to discuss issues with healthcare professionals. Such skill development may not necessarily result in decreased utilization, but may result in more efficient and effective use of services which may take time to develop and stabilise.

Both, the Stanford Chronic Disease Self-Management Programme and the UK Expert Patient Programme are either peer-led or have a combination of professional and peer led sessions, whereas OPTIMAL is completely professionally led by occupational therapists with no peer leadership roles. Although the benefits of peer support are frequently reported, such as sharing of experiences and feelings of empowerment, there are potential adverse effects, such as how peer support can increase feelings of isolation if participants perceive their peers as having dissimilar lifestyles [49]. Individual negativity in the group can adversely impact on group dynamics and increase the potential for negative social comparisons [49]. Another identified disadvantage of peer-led groups is a lack of control over interactions in the group, providing no guarantee that the support given is beneficial [50]. Professionally-led groups can address these potential difficulties while also harnessing the potential benefits of peer support and interactions, and have also been found to be effective in other areas such as cardiac and pulmonary rehabilitation [51-53].

As outlined in the results, low attendance was an issue and a number of participants missed sessions. In other self-management programme studies the number of participants per group ranged from 8 to 15 [13,54]. Initially the aim in this study was to recruit a minimum of 10 participants for each group, however due to lower than anticipated recruitment rates and low attendance six or less participants consistently attended each of the three separate programmes. The time between baseline data collection and group commencement (up to eight weeks later) may have been a contributing factor to non-attendance. Of the 26 participants in the intervention group, 20 (77%) attended one session and 16 (62%) attended 3 or more sessions. A Cochrane review found similar results, finding that between 51% and 87% of lay-led self-management programme participants attended at least half the self-management programme sessions, and between 8% and 29% never attended any sessions [55]. A larger study is needed to assess the effect of attendance on outcomes, and examine differences based on age, gender and whether participants are engaged in paid employment.

This was a pragmatic feasibility trial that included patients commonly seen in clinical practice and referral to the study was designed to reflect current referral pathways for additional primary care services. However, due to the relatively short timeframe involved in undertaking the study, this may have led to some selection bias as clinicians may have been more likely to refer patients that were better known to them. On the other hand, this does reflect the pragmatic nature of the study as this is how referral would operate were the OPTIMAL intervention to be normalised into routine practice. A further limitation of this research is its small sample size and short follow-up period. Hence, it is not possible to ascertain whether benefits were sustained over time. Additionally, study participants were aware of their group allocation and some participant ascertainment bias may have occurred. A further limitation relates to the lack of blinding of outcome assessors. This is challenging in trials testing complex interventions as participants are likely to talk about their intervention experiences but this could be addressed in a larger trial with more resources for independent data collection. A strength of the OPTIMAL intervention is that it requires minimal training and resources to deliver and could be used in any primary care setting where occupational therapists are available. It is also possible that other primary care professionals could lead this programme with appropriate training and this would improve OPTIMAL generalizability across settings.

## Conclusion

This study has provided preliminary evidence that OPTIMAL is effective in improving a range of outcomes for individuals with multimorbidity and contributes towards the evidence base on the effectiveness of interventions for people with multimorbidity. We now need a definitive trial that will test the cost effectiveness and sustainability of the OPTIMAL programme over a longer time period and across a wider range of primary care settings.

### Availability of supporting data

Secondary analyses are still ongoing but the data from this study will be made available on an open access repository when these analyses are complete. In the meantime, please contact the corresponding author if you have any queries regarding the supporting data.

## Abbreviations

Qol: Quality of life
ADL: Activities of daily living
COPM: Canadian occupational performance measure
NEADL: Nottingham extended activities of daily living
HADS: Hospital anxiety and depression scale
SSE: Stanford chronic disease self-efficacy 6-item scale
HeiQ: Health education impact questionnaire
GAS: Goal attainment scaling
HCU: Healthcare utilisation
